# Back to Basics: The Diagnostic Value of a Complete Blood Count in the Clinical Management of COVID-19

**DOI:** 10.3390/diagnostics14171933

**Published:** 2024-09-02

**Authors:** Anwar A. Sayed

**Affiliations:** Department of Basic Medical Sciences, College of Medicine, Taibah University, Madinah 42353, Saudi Arabia; dsayed@taibahu.edu.sa; Tel.: +966-014-861-8888

**Keywords:** CBC, COVID-19, diagnostics, mortality, pathophysiology, principal component analysis, Saudi Arabia

## Abstract

Since the beginning of the COVID-19 pandemic, scientists have struggled significantly to understand the complexity of COVID-19 pathophysiology. COVID-19 has demonstrated a notoriously unpredictable clinical course. This unpredictability constituted a significant obstacle to clinicians in predicting the disease course among COVID-19 patients, more specifically, in predicting who would develop severe cases and possibly die from the infection. This brief report aims to assess the diagnostic value of using a complete blood count (CBC) and applying high-dimensional analysis, i.e., principal component analysis (PCA), on it to differentiate between patients with mild and severe COVID-19 infection. The data of 855 patients were retrieved from multiple centres in Saudi Arabia. Descriptive statistics, such as counts, percentages, and medians (interquartile ranges) were used to describe patients’ characteristics and CBC parameters. Analytical statistics, such as the Mann–Whitney *U* test, were used to compare between survivors and non-survivors. PCA was applied using the CBC parameters, and the results were compared between survivors and non-survivors. Patients in this study had a median age of 41, with an almost equal ratio of men to women. Most participants were Saudis, and non-survivors were 13.22% of the total cohort. The median values of all CBC indices were within reference ranges; however, some statistically significant differences were observed between survivors and non-survivors. Non-survivors had lower hemoglobin levels and lower hematocrit, lymphocyte, and eosinophil counts but higher WBC and neutrophil counts compared to survivors. PCA on the CBC results of survivors yielded a significantly different profile than non-survivors, indicating the possibility of its use in the context of COVID-19. The diagnostic value of CBC in the clinical management of COVID-19 should be utilized in clinical guidelines for managing COVID-19 cases.

## 1. Introduction

The recent novel coronavirus disease 2019 (COVID-19) pandemic has hit the world by storm [1]. Given its morbidity and mortality, the pandemic prompted countries to take progressive public measures, which seemed to limit its impact on these societies [2,3,4,5,6,7,8].

The pathogenesis of COVID-19 was initially thought to be respiratory [9]. However, more emerging evidence describes its extrapulmonary course, including immunological and hematological abnormalities [10,11]. The more scientists uncover aspects of COVID-19 pathophysiology, the more we can determine factors contributing to and possibly predicting COVID-19 severity and subsequent mortality.

High-dimensional analysis, e.g., principal component analysis (PCA), is an analytical tool used to visualize large datasets and flatten them to demonstrate the linkage between the different variables [12,13]. In biological sciences, it could be used on large-scale genome-wide data [14], which allowed for its use in the context of COVID-19 on genomic sequence studies [15], as well as for immunological single-cell profiling and analysis [16,17].

In this study, the value of PCA is assessed in differentiating the pathophysiology of COVID-19 patients, specifically, between survivors and non-survivors.

## 2. Materials and Methods

### 2.1. Study Design and Setting

This is a retrospective study of patients with COVID-19 across three hospitals in Saudi Arabia. We included in the study 855 patients whose hospital admission was based on a suspicion of COVID-19. A nasopharyngeal swab was tested using quantitative polymerase chain reaction (qPCR) to confirm the diagnosis of COVID-19. Individuals partaking in this research were hospitalized from June 2020 to December 2021 according to the Saudi Ministry of Health guidelines for admitting COVID-19 cases [18]. Briefly, these hospitalized individuals had one of the following signs: clinically proven pneumonia, age over 65, low oxygen saturation below 94% on a pulse oximeter while not using supplemental oxygen, acute respiratory distress syndrome, chronic lung or renal disease, history of other health conditions, or severe obesity with a BMI of 40 or higher. This study included patients who were admitted and diagnosed with positive/confirmed COVID-19. Patients with negative COVID-19 PCR tests or unconfirmed/missing information were excluded from this study.

The treatment of these hospitalized patients was uniform across all three hospitals, adhering to the national COVID-19 management protocol set by the Saudi MoH for Patients Suspected of/Confirmed with COVID-19 [18]. Intensive care unit (ICU) admission was reserved for those patients with suspicion of/confirmed severe COVID-19, based on the Saudi MoH Protocol for Patients Suspected of/Confirmed with Severe COVID-19 [18]. Clinically, those patients with severe COVID-19 showed signs of pneumonia, e.g., fever, cough, shortness of breath, in addition to one of the following conditions: breathing rate exceeding 30 breaths per minute, oxygen saturation below 93% on a pulse oximeter while breathing regular air, or experiencing severe respiratory distress. Patients were divided into two categories based on the outcome of their admission: either survivors, defined as those who recovered from COVID-19 infections and were eventually discharged from the hospital, and non-survivors, i.e., those who died due to COVID-19 infection.

Each patient underwent a range of lab tests, with some being repeated during their time in the hospital. For this study, only the initial readings from their investigations were included to maintain consistency in the parameters being compared, which were measured upon admission. Patients’ venous blood samples were gathered for analysis as a component of their hospital care. Venous blood samples underwent complete blood count testing with the Mindray BC-3200 Auto Hematology Analyzer manufactured by Shenzhen Mindray Bio-Medical Electronics Co. in Shenzhen, China. The gathered data were derived from a CBC test, which covers RBC count, hemoglobin level, MCV, MCH, platelet count, WBC count, neutrophil count, lymphocyte count, monocyte count, and eosinophil count.

### 2.2. Statistical Analysis

Gender and nationality were depicted using absolute values and percentages for categorical data representation. Descriptive statistics were utilized to represent numerical data, like red cell blood count and platelet count, based on their distribution, as determined by the Shapiro–Wilk test. Parametric data were described using means and standard deviations, whereas nonparametric data were described using medians and interquartile ranges. Comparison between two parametric and nonparametric groups were compared using either Student’s *t*-tests or Mann–Whitney *U* tests, respectively. Statistical significance was denoted at a *p* value of less than 0.05. Exploratory principal component analysis (PCA) was performed using all the parameters generated from a standard CBC test, namely: RBC count, MCV, MCH, platelet count, WBC count, neutrophil count, lymphocyte count, monocyte count, and eosinophil count. PCA was performed separately on parameters from survivors and non-survivors [15,19]. The two principal components (PCs) with the highest percentages of variance (of 60% variance) are reported and described in the results section. Patients with more than 10% missing data were excluded from the analysis [14]. Data analysis and figure generation were carried out using GraphPad Prism Version 10.2.2 for Windows. 

### 2.3. Ethical Considerations

This study’s collected data did not include personal or sensitive patient information. Measures were taken to preserve and safeguard the collected information via storage on a password-protected drive backed up on a password-protected server. This study was conducted following the declaration of Helsinki and ethically approved by the Taibah University College of Medicine Research Ethics Committee no. TU-21-010. Given the retrospective nature of this study, patients’ consent was waived by the approving IRB ethics committee.

## 3. Results

A total of 855 patients were included in this study. Their median age was 41, with a calculated interquartile range between 27 and 57 years old. In other words, most of the study participants are adults who are not elderly. 

The study participants had an almost equal gender distribution, with 426 males and 429 females. Almost 65% of the study participants were Saudis (n = 550), whereas the remainder were non-Saudis (n = 305). Of the study participants, over 13% of the participants died due to COVID-19 (n = 113), whereas the remaining study participants survived the infection (n = 742).

The participants’ hematological parameters were investigated using a CBC, and their blood profiles were demonstrated. The median value of the participants’ red blood cell (RBC) count was 4.74 (4.35–5.13) × 10^6^/mL, falling within normal ranges for males and females. Similar to the RBC count, the median hemoglobin value was 13.40 (11.99–14.60) g/dL, falling within the normal range for males and females. Other parameters, such as MCV, MCH, platelet count, WBC count, and differential leukocyte count, were also evaluated. Interestingly, they were all within the reference range for the entire cohort. The differences in the CBC parameters between survivors and non-survivors are summarized in Table 1.

**Figure 1:**
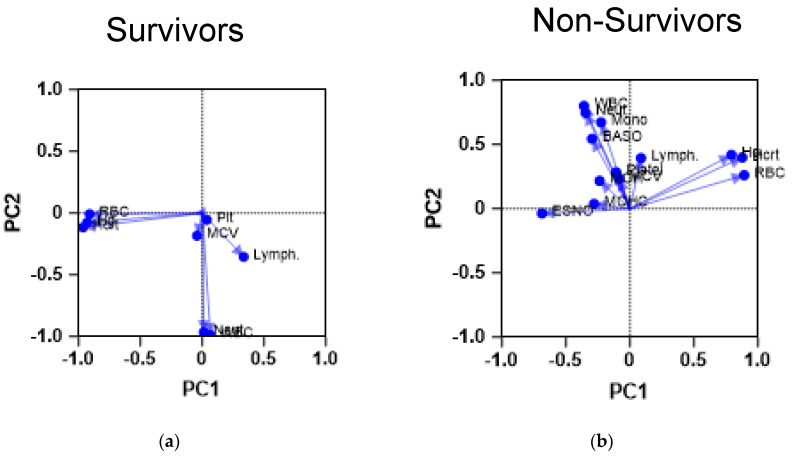
Principal component analysis of CBC parameters of the study cohorts. The figures demonstrate the results of a PCA performed on the CBC parameters of the patients. The scatter plots demonstrate the PCA profile of both (**a**) survivors (n = 742) and (**b**) non-survivors (n = 113). The first principal component (PC1) is plotted on the *x*-axis and accounts for 35% of the total variance, while the second principal component (PC2) is plotted on the *y*-axis, explaining 25% of the variance. Baso: basophil count; ESNO: eosinophil count; Hcrt: hemotocrit level; Hg: hemoglobin level; Lymph: lymphocyte count; MCV: mean corpuscular volume; Mono: monocyte count; Neut: neutrophil count; Plt: platelet count; RBC: red blood cell count; WBC: white blood cell count.

The next step was to examine the CBC results of survivors and non-survivors using PCA to assess whether they would yield similar results. Expectedly, the PCA analysis of both cohorts yielded different CBC profiles, as shown in Figure 1.

Survivors demonstrate a CBC profile, which shows most of the RBC parameters compiled together, whereas platelet count and lymphocyte count seem to be compiled close to each other. Such proximity may indicate the importance of the previously described indicator, the systematic immunoinflammatory index. On the other hand, patients with severe COVID-19 demonstrated a different profile in which white blood cell parameters were separate from the red blood cell indices, except for lymphocyte counts, which were closer to platelet counts. This PCA finding could point to a link between these parameters in the context of COVID-19.

## 4. Discussion

COVID-19 infection and, in particular, its pathophysiology, has posed a dilemma to the scientific community. However, the sheer number of studies on this subject, using a wide range of assessment tools, from clinical observations to very sophisticated diagnostic techniques, has started to untangle its elements. 

Early reports described SARS-CoV-2 as a respiratory virus affecting the lung alveoli and gaining entry into them through its spike protein [21,22]. Subsequently, this infection, due to its novel nature and the lack of previous exposure to vaccination, will cause severe acute respiratory distress syndrome, primarily due to diffuse alveolar damage leading to severe deterioration in respiratory function [23,24]. Nevertheless, COVID-19 infections seemed to extend beyond the lungs and carry extrapulmonary features, which have been described and used clinically.

**Figure 2:**
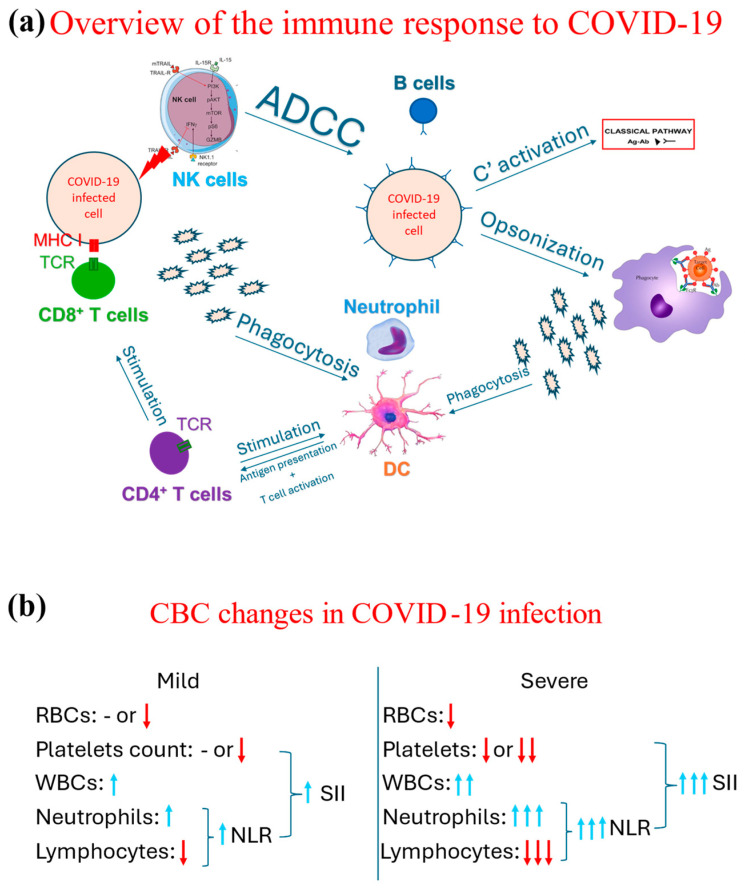
Schematic representation of overview of the immunological response to COVID-19 and the associated changes observed in a CBC in cases of COVID-19. (**a**) Demonstrates an overview of the immune response, both the innate (through phagocytes, NK cells, and complement system through its classical pathway triggered by the antigen-antibody complext) and adaptive immune response (T and B cells). (**b**) Contrasts the changes associated with the CBC indices between mild and severe COVID-19 cases, in which blue arrows mean increase and red arrow refer to a decrease, while the number of arrows highlight the degree of such an increase or decrease. Ab: Antibody, ADCC: antibody-dependent cytotoxicity, Ag: Antigen, C’: complement, DC: dendritic cells, MHC: major histocompatibility complex receptor, NK: natural killer, NLR: neutrophil-to-lymphocyte ratio; RBC: red blood cell; SII: systemic immunoinflammatory index; TCR: T cell receptor, WBC: white blood cell.

Several hematological changes have been described as a part of the COVID-19 pathophysiology. The COVID-19 infection drives a proinflammatory response in which both arms of the immune system, innate and adaptive, are involved, as demonstrated in Figure 2a. As part of the initial, i.e., innate, immune response, neutrophils are one of the earliest cells to respond [25], increasing their count. Such an increase directly correlates with the case severity; i.e., the more severe the case is, the more that the neutrophil count increases [26,27]. Neutrophils and other phagocytes phagocytose the virus, breaking it into smaller antigens. These smaller antigens are then released into the circulation and taken up by dendritic cells to present them to helper CD_4_ T cells. The complement system is also involved in the immune response to COVID-19 [28,29], even though it is suggested that the complement system may lead to hypercoagulability and collateral damage to other non-respiratory organs [30]. 

Both humoral (antibody-mediated) and cellular (T cell-mediated) responses are involved in tackling COVID-19 infection [31,32,33]. However, it was noted that overall lymphocytic count seemed to decrease along with the severe cases; i.e., the more severe the case, the lower the lymphocytic count is [34,35]. This reduction is part of an immunological exhaustion observed in the context of COVID-19 [36,37].

The continuous effort of researchers and the replicability of these findings across populations has prompted clinicians and scientists to propose neutrophil-to-lymphocyte ratio (NLR) as a predictor of COVID-19 severity. NLR is calculated by dividing the absolute counts of neutrophils by the absolute count of lymphocytes. Hence, the higher the NLR is (by the increase in neutrophil count and further reduction of lymphocyte count), the more likely it is that the patient is suffering from severe COVID-19 [27]. NLR was subsequently considered as an indirect mortality predictor [38,39,40]. 

Many proinflammatory cytokines are released as part of the immunological response against COVID-19, creating a toxic milieu for other hematological cells [41,42]. For example, the proinflammatory environment could lead to an autoreactive hemolytic response targeting red blood cells, subsequently causing anemia [43,44]. Furthermore, platelets are also involved in the pathogenesis of COVID-19. Their involvement was proposed to be an activation leading to thrombosis and coagulation abnormalities [45] or being destroyed by autoantibodies secondary to SARS-CoV-2 infection [46]. Based on the multicellular involvement in the pathophysiology of COVID-19, it was no surprise that more indicators were developed, such as the systematic immunoinflammatory index (SII) [47,48]. This indicator is calculated based on the NLR and platelet count and is used to predict COVID-19 severity and mortality [20,49,50]. These changes are summarized in Figure 2b.

The use of PCA in the context of COVID-19 has been previously described, mainly in the context of genomic studies [15]. Subsequently, it has been used to cluster COVID-19 patients into categories based on multiple variables, including age, hematological indices, and other biological markers [51]. However, these previously reported usages of PCA shed light on its value in the context of large datasets generated from investigations that may not be widely available. This brief report is the first to report the use of PCA on parameters generated from a CBC test, a tool that is rapid, inexpensive, and widely available [52]. The use of PCA could demonstrate separate profiles for survivors and non-survivors, in line with previous studies [51,53]. In fact, the PCA has the capability to dissect the complexity of COVID-19 pathophysiology, demonstrated in Figure 2, and, rather, shows the interacting elements (cells) on an individualized level. Such an approach could further strengthen the role of advanced machine learning techniques in medicine [54], which is a landmark of this era. This brief report adds to the existing literature the suggestion that modern approaches, i.e., PCA, could indeed be incorporated into daily medical practice and be applied to other novel emerging conditions such as human monkeypox. 

While this brief report offers valuable new perspectives to the existing scientific literature, it does have some limitations. Firstly, due to the retrospective cross-sectional nature of this study, it is susceptible to sampling bias [55]. Also, other factors, which are usually found and observed upon clinical examination in the PCA, were not taken into account. These include vital signs (temperature, blood pressure, and oxygen saturation) or symptoms (cough and shortness of breath); these factors may be significant indicators of COVID-19 severity and of subsequently mortality. Finally, this research solely concentrated on values collected and derived from a standard CBC. Nonetheless, the significance of other blood markers, like C-reactive protein (CRP) and interleukin (IL)-6, in forecasting COVID-19 severity and related mortality remains essential [56,57].

## 5. Conclusions

The pathophysiology of COVID-19 presented a challenge to many worldwide in its interpretation and use in clinical practice. Nevertheless, studies have demonstrated the invaluable importance of indicators such as NLR and SII, derived from a CBC, as practical tools for predicting COVID-19 severity and mortality. The present study further sheds light on the importance of applying PCA on CBC results. This importance is demonstrated in the differential profiles for those who may experience severe COVID-19 compared to milder cases, which is of great clinical significance and could direct the treatment course. Such a finding may be helpful in clinical practice, mainly if automatically generated with each CBC report. It also paves the way for future use in emerging conditions, e.g., in human monkeypox.

## Figures and Tables

**Table 1:** Comparison of patients’ lab investigations between survivors and non-survivors.

Investigation (Unit)	Survivors (n = 742)	Non-Survivors (n = 113)	*p* Value
RBC count (×10^6^/mL)	4.76 (4.37–5.14)	4.60 (4.09–5.08)	0.09
**Hemoglobin (g/dL)**	**13.50 (12.20–14.70)**	**12.88 (11.60–14.35)**	**0.007**
**Hematocrit (%)**	**41 (37.30–44.20)**	**39.70 (35.70–43.30)**	**0.046**
MCV (fl)	86.20 (81.70–89.80)	86.70 (80.90–89.90)	0.73
MCH (pg)	28.70 (26.90–30)	28.10 (26.40–29.90)	0.23
Platelet count (×10^6^/mL)	228.5 (185–294)	235 (160.9–285)	0.46
**WBC count (×10^3^/mL)**	**5.96 (4.45–8.56)**	**8.33 (5.78–11.60)**	**<0.0001**
**Neutrophil count (×10^3^/mL)**	**3.93 (2.57–6.63)**	**6.16 (4.16–9.86)**	**<0.0001**
**Lymphocyte count (×10^3^/mL)**	**1.13 (0.74–1.66)**	**0.76 (0.53–1.25)**	**<0.0001**
Monocyte Count (×10^3^/mL)	0.33 (0.23–0.48)	0.33 (0.20–0.53)	0.32
**Eosinophil count (×10^3^/mL)**	**0.04 (0.01–0.08)**	**0.02 (0.00–0.06)**	**0.002**

All values are expressed as medians (interquartile range). Comparisons between survivors and non-survivors were made using the Mann–Whitney *U* test based on their nonparametric distribution. Values written in bold denote statistically significant differences between survivors and non-survivors. The results demonstrated in this study are a secondary analysis of data presented in a previous study [20].

## Data Availability

The data presented in this study are available on reasonable request from the corresponding author.
